# *Clec7a* drives gut fungus-mediated host lipid deposition

**DOI:** 10.1186/s40168-023-01698-5

**Published:** 2023-11-25

**Authors:** Jie Ma, Miao Zhou, Zehe Song, Yuankun Deng, Siting Xia, Yunxia Li, Xingguo Huang, Dingfu Xiao, Yulong Yin, Jie Yin

**Affiliations:** 1 College of Animal Science and Technology, Hunan Agriculture University, Changsha, 410128 China; 2 College of Animal Science and Technology, Guangxi University, Nanning, 530004 China; 3 Institute of Subtropical Agriculture, Chinese Academy of Sciences, Changsha, 410125 China

**Keywords:** Gut fungi, Lipid metabolism, *Clec7a*

## Abstract

**Background:**

Compared to that of bacteria, the role of gut fungi in obesity development remains unknown.

**Results:**

Here, alterations in gut fungal biodiversity and composition were confirmed in obese pig models and high-fat diet (HFD)-fed mice. Antifungal drugs improved diet-induced obesity, while fungal reconstruction by cohousing or fecal microbiota transplantation maintained the obese phenotype in HFD-fed mice. Fungal profiling identified 5 fungal species associated with obesity. Specifically, *Ascomycota*_sp. and *Microascaceae_*sp. were reduced in obese mice and negatively correlated with fat content. Oral supplementation with fungi was sufficient to prevent and treat diet-induced obesity. *Clec7a*, which is involved in fungal recognition, was highly expressed in HFD-fed mice. The *Clec7a* agonist accelerated diet-induced obesity, while *Clec7a* deficieny in mice resulted in resistance to diet-induced obesity and blocked the anti-obese effect of antifungal drugs and fungi.

**Conclusions:**

Taken together, these results indicate that gut fungi/*Clec7a* signaling is involved in diet-induced obesity and may have therapeutic implications as a biomarker for metabolic dysregulation in humans.

Video Abstract

**Supplementary Information:**

The online version contains supplementary material available at 10.1186/s40168-023-01698-5.

## Background

Over recent decades, the proportion of overweight or obese individuals, especially children, has risen rapidly in the world, in part due to increased food intake, decreased energy expenditure, and lifestyle changes [1–3]. Obesity is generally accompanied by other metabolic diseases (i.e., diabetes, cardiovascular diseases, hypertension, and nephropathy) [4], which results in an enormouse economic burden [5]. Thus, there is significant interest in studying the mechanisms underlying obesity development, as this may lead to more effective treatment options for obesity and enable individuals to improve their health.

The gut microbiota is implicated in obesity occurrence and development [6–10], and probiotic is now widely used to alter the gut microbiota as part of a treatment strategy for obesity [11–14]. Knowledge on the interaction between the gut microbiota and host metabolism mainly focuses on bacteria while neglecting the role of gut fungi due to their lower abundance. However, the observation that many gut fungi are strongly correlated with host health has stimulated interest in further research on gut mycobiota. Notably, mycobiota dysbiosis was characterized in patients with inflammatory bowel disease (IBD), and various fungi that elicit strong inflammatory cytokine production, exacerbating colitis, have been identified [15–18]. In obese patients, the fecal mycobiome is disturbed compared to nonobese subjects [19], indicating a potential role for fungi in the development and manipunation of obesity. To date, the mechanisms by which gut fungi contribute to diet-induced obesity remain elusive. It has been proposed that *Clec7a*, a member of the C-type lectin receptor (CLR) family that encodes the dectin1 protein and recognizes β-glucans at fungal cell walls [20], might be a key factor in the development of fungus-associated obesity, as *Clec7a* expression was upregulated in adipose tissues, while dectin-1 antagonist treatment was observed to improve glucose homeostasis [20].

Here, we observed fungal alterations in obese pig models and high-fat diet (HFD)-fed mice. The use of antifungal drug treatment to disrupt gut fungi but bot bacteria protected against diet-induced obesity and improved glucose tolerance, insulin sensitivity, and energy expenditure in HFD-fed mice. The anti-obese effect was blocked in fungal reconstructed mice by cohousing or fecal microbiota transplantation. High-throughput rDNA sequencing analysis identified 5 associations between fecal fungi and fat content. Surprisingly, oral supplementation of mice with the negatively correlated fungi (*Ascomycota_*sp. and *Microascaceae_*sp.) was sufficient to prevent and treat diet-induced obesity. Moreover, in line with the hypothesis that *Clec7a* might be associated with obesity, we observed that *Clec7a* KO mice were resistant to diet-induced obesity. Finally, we found that *Clec7a* deficiency blocked the anti-obese effect of gut fungi in mice given antifungal and fungi-mediated therapy. These results suggest that in addition to bacteria, a healthy fungal microbiome is also important for lipid metabolism, and that gut fungi/*Clec7a* may have therapeutic implications as a biomarker for metabolic dysregulation in humans.

## Results

### Gut fungal species are altered in obese pigs and mice

Compared with lean pigs, such as Yorkshire, Landrace, and Duroc pigs, Chinese native pig breeds are generally classified as fatty animal models. We analyzed fecal fungi from 150-day-old obese Shaziling pigs and lean Yorkshire pigs housed on different pig farms and found that *α*-diversity (Chao1 index) and *β*-diversity (PCoA plot) were markedly different in obese Shaziling pigs compared to Yorkshire pigs (Fig. S1A). To exclude the possibility that differences in fungal diversity were due to differences in diet and feeding environment, we further sequenced the fecal fungi in obese Ningxiang pigs (55.63 ± 1.47 kg), and lean Duroc Landrace-Yorkshire (DLY) pigs (109.25 ± 2.35 kg) fed the same diet in the same environment and found a similar reduction in diversity in Ningxiang pigs (Fig. S1B). Interestingly, this phenomenon was not observed between HFD-fed mice (28.84 ± 0.41 g) and control mice (31.03 ± 0.38 g) (Fig. S1C). At the phylum level, Ascomycota was the most abundant phylum in all three animal models. LDA analysis revealed different markers in Shaziling and Ningxiang pigs and HFD-fed mice (Fig. S1D–F). Together, these data point to a representative gut fungal community in host-related lipid metabolism or diet-induced obesity.

### Fungi deficiency protects mice against diet-induced obesity

**Fig. 1:**
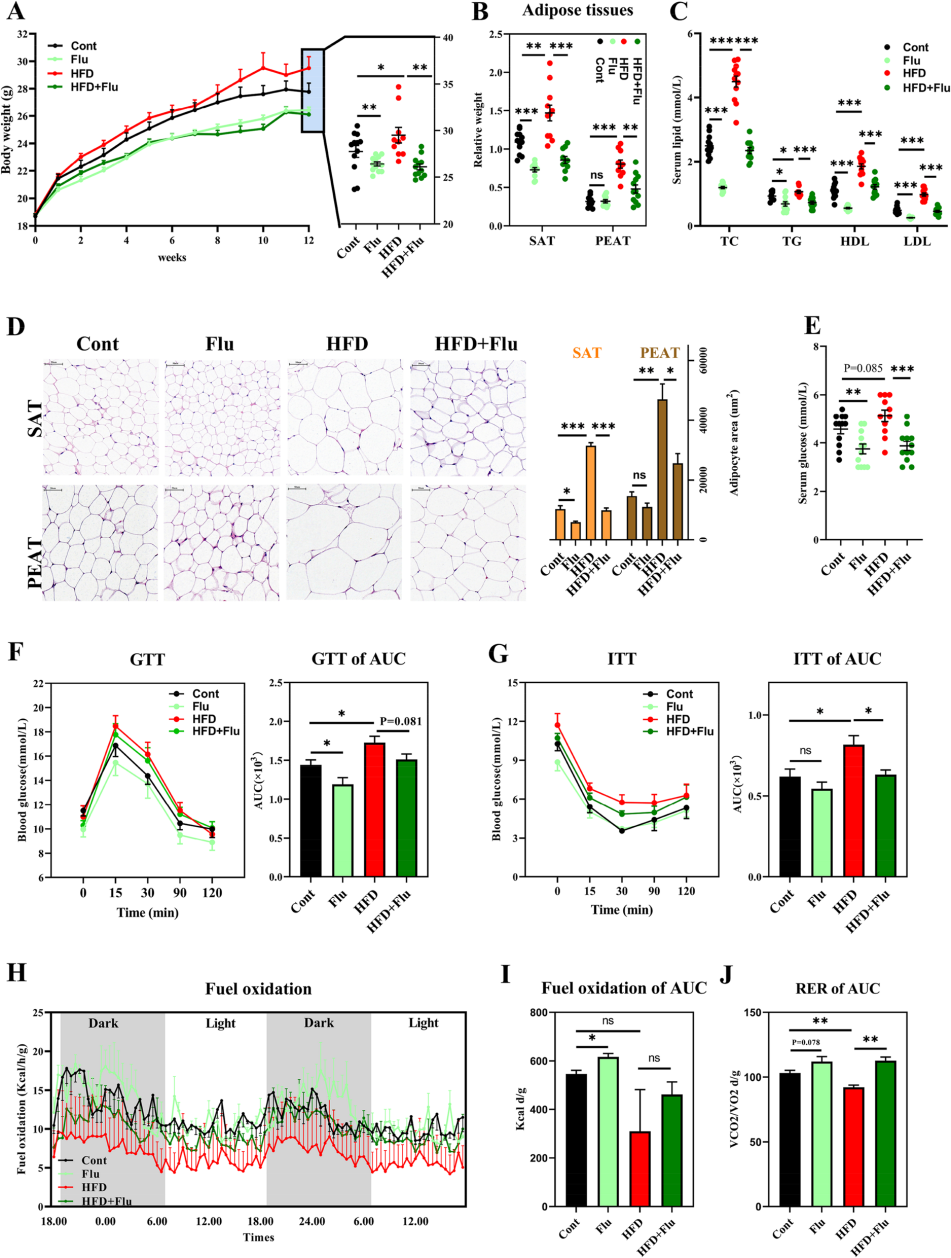
Fungi deficiency protects mice against diet-induced obesity. **A**–**E** Body weight gain (**A**), the relative weight of subcutaneous adipose tissue (SAT) and perirenal adipose tissue (PEAT) (%) (**B**), serum lipids (**C**), adipocyte size and area of SAT and PEAT (**D**), and serum glucose (**E**) in fluconazole (Flu)-treated mice. C57BL/6 mice (6 weeks old, male) were challenged with HFD, or fluconazole lasted for 12 weeks (*n* = 10–13). SAT and PEAT were staining with HE, and the adipocyte size and area were examined using Case Viewer and ImageJ software (× 400). **F** and **G** GTT and ITT and their area under the curve (AUC). C57BL/6 mice were fasted for 10–12 h and subjected to GTT test with intraperitoneal injection of glucose (1 g/kg body weight) and then for ITT test with intraperitoneal injection of insulin (0.75 U/kg body weight) 4 h after restriction. Glucose levels were measured at 15, 30, 60, 90, and 120 min (*n* = 8). **H**–**J** Metabolic parameters (O_2_, CO_2_, and energy consumption) of HFD- and fluconazole-treated mice were recorded in an International FOXBOX.™ field oxygen analysis system for 48 h, and then the alibrated data were used for calculating the fuel oxidation and respiratory exchange ratio (RER) (*n* = 8). Differences among the groups were compared using Student’s *t*-test. *p < 0.05; **p < 0.01; ***p < 0.001; ns *p* > 0.05

To validate the role of gut fungi in obesity development, we next generated a pseudo fungus-deficient mouse model in which gut fungi were eliminated by oral fluconazole [21, 22]. Consistent with the phenotype of germ-free mice [23, 24], these fungus-deficient mice (fluconazole treated for 12 weeks) had lower body weight and size relative to control (Fig. 1A). The relative weights of subcutaneous adipose tissue (SAT) and perirenal adipose tissue (PEAT) were markedly lower in fungus-deficient mice fed a HFD (Fig. 1B). HE staining of SAT and PEAT showed reduction in adipocyte size and area in fungi-deficient mice (Fig. 1D). No changes were observed in abdominal adipose tissue (AAT) or epididymal adipose tissue (EAT) between control and fungus-deficient mice (data not shown). Similarly, both male and female HFD-fed mice receiving a 16-week fluconazole treatment presented with lower body weight, SAT, AAT, and PEAT weight and a downregulaition of dectin-1, a fungal sensor (Fig. S2), which further excluded the gender influence of sex.

Serum lipid profiling showed a robust decrease in the concentrations of total cholesterol (TC), triglyceride (TG), high-density lipoprotein (HDL), and low-density lipoprotein (LDL) in fungus-deficient mice that were or were not fed a HFD (Fig. 1C). Fungal deficiency for 12 weeks markedly reduced serum glucose levels in control or HFD-fed mice (Fig. 1E), suggesting a role of gut fungi in improving glucose tolerance. Thus, a glucose tolerance test (GTT) and insulin tolerance test (ITT) were performed in 16-week fluconazole-treated mice. Glucose tolerance was slightly improved in fungus-deficient mice (Fig. 1F and G). Although fluconazole treatment did not alter insulin concentrations as observed with glucose, fluconazole exposure significantly improved insulin tolerance in HFD-fed mice (Fig. 1G). These data demonstrate that mice with fungal deficiency exhibited improved glucose tolerance, possibly due to enhanced insulin sensitivity.

Using metabolic chambers, we determined the energy expenditure and respiratory exchange ratio (RER) of HFD- and fungus-deficient mice. All mice had similar fuel oxidation patterns, rapidly rising at night and then falling to ~ 7–11 during the light cycle (Fig. 1H). While the HFD-fed mice had a lower fuel oxidation curve, both fungi-deficient mice fed control or HFD diets exhibited higher curves. We further calculated fuel oxidation and RER per day and found that fungal deficiency enhanced fuel oxidation and RER in HFD-fed mice (Fig. 1I and J).

### Fungal reconstruction maintains diet-induced obesity

**Fig. 2:**
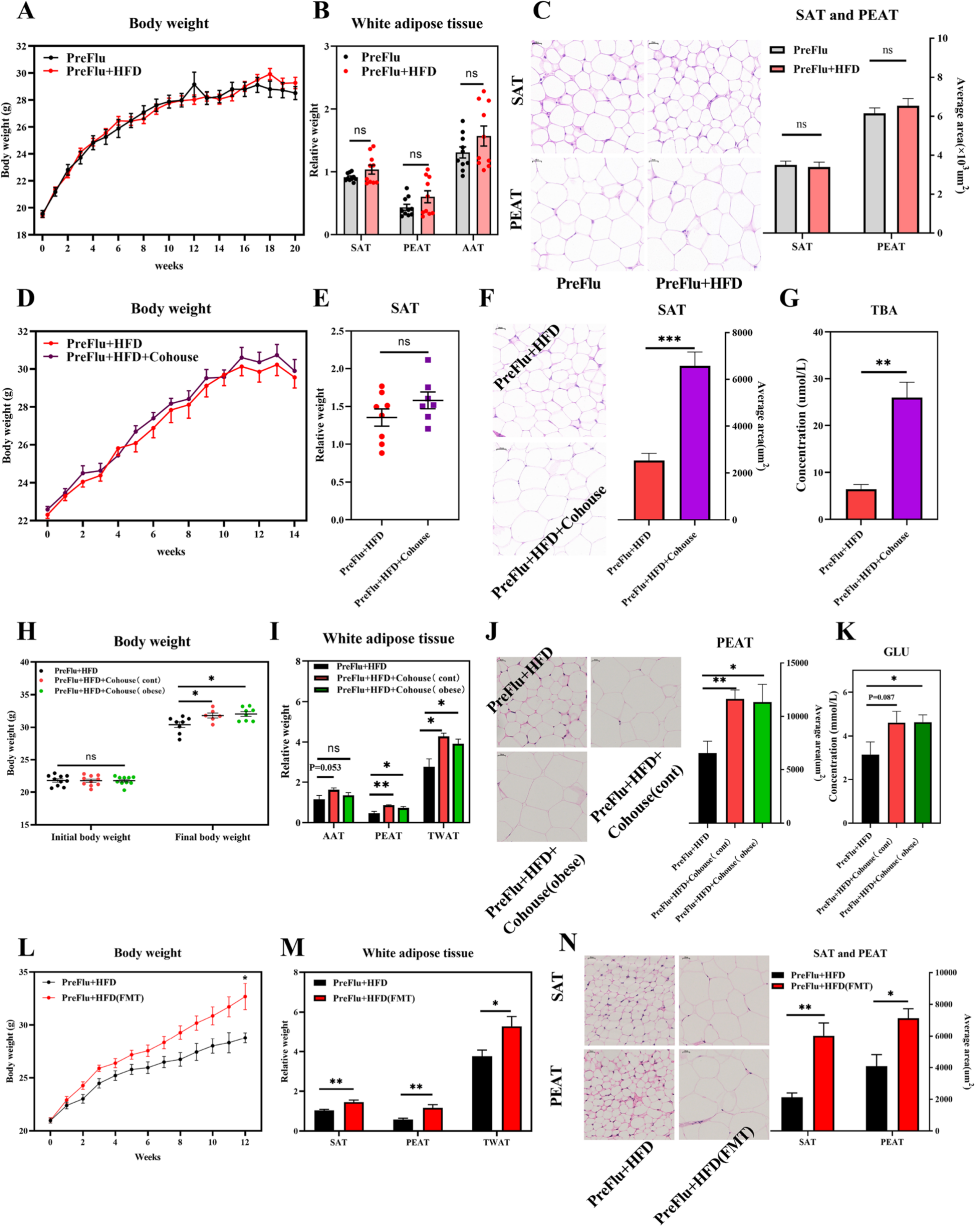
Fungal reconstruction maintains diet-induced obesity. **A**–**C** Body weight gain (**A**), the relative weight of white adipose tissues, and adipocyte size and area of SAT and PEAT (HE staining, × 400) in flu-pretreated mice. C57BL/6 mice (6 weeks old, male) received 1-week fluconazole (PreFlu) and then fed HFD (PreFlu + HFD) lasted for 20 weeks (*n* = 10). **D**–**G** Body weight gain (**D**), the relative weight of SAT (**E**), adipocyte area of SAT (HE staining, × 400), and serum bile acid (TBA) (**G**) in flu-pretreated mice cohousing with control mice. C57BL/6 mice (7 weeks old, male) with PreFlu and HFD exposure were cohoused with (PreFlu + HFD + cohouse) or without healthy mice for 14 weeks to rebuilt gut fungal communities (*n* = 10). **H**–**K** Body weight (**H**), the relative weight of white adipose tissues (**I**), adipocyte area of PEAT (HE staining, × 400), and serum glucose (**K**) in Flu-pretreated mice cohousing with healthy or obese mice. C57BL/6 mice (7 weeks old, male) with PreFlu and HFD exposure were cohoused with obese mice (31.67 ± 0.37 g) for 18 weeks to rebuilt gut fungal communities (*n* = 7–10). **L**–**N** Body weight gain (**L**), the relative weight of white adipose tissues (**M**), and adipocyte area of SAT and PEAT (HE staining, × 400) in Flu-pretreated mice with fecal microbiota transplantation (FMT). C57BL/6 mice (7 weeks old, male) with PreFlu and HFD exposure were transplanted with fecal microbiota from healthy donors (PreFlu + HFD (FMT)) for 16 weeks to rebuilt gut fungal communities (*n* = 4–10). Differences among the groups were compared using Student’s *t*-test. *p < 0.05; **p < 0.01; ***p < 0.001; ns *p* > 0.05

Next, we investigated the preventive effect of short-term antifungal drug exposure on HFD-induced obesity development. Mice pretreated with fluconazole for 1 week were resistant to HFD-induced obesity, and this trend lasted for 20 weeks (Fig. 2A). In addition, the relative weight of white adipose tissues (Fig. 2B) and the adipocyte area (Fig. 2C) did not increase in HFD-fed mice pretreated with fluconazole. Together, these data indicate that 1 week of antifungal fluconazole pretreatment also protected mice from HFD-induced body weight gain and fat deposition.

Mice pretreated with fluconazole were further cohoused with control mice for 14 weeks to test whether fungal community reassembly maintained diet-induced obesity. Although reconstruction of the fungal community did not increase body weight (Fig. 2D), SAT (Fig. 2E), or PEAT weight (data not shown), the adipocyte size and area of SAT significantly increased in cohoused mice (Fig. 2F). Bile acid is highly associated with lipid absorption and deposition, and higher serum bile acid levels were also observed in cohoused mice (Fig. 2G). Although body weight was not markedly increased (Fig. 2H), accelerated lipid deposition in the relative weight of PEAT and total white adipose tissue (Fig. 2I and J) and increased serum glucose (Fig. 2K) were also observed in fluconazole-pretreated mice cohoused with obese donors for 18 weeks. Cohousing fluconazole-pretreated mice with control mice led to the restored gut fungi resembling that of the donor (Fig. S3A, B). A similar observation was made in mice cohoused with obese mice (Fig. S3C, D). Transplanting normal fecal microbiota further confirmed the role of gut fungi in obesity, as fluconazole-pretreated mice showed improvements in body weight gain and lipid deposition after receiving normal fecal microbiota from healthy donors (Fig. 2L–N). Specifically, transplanting donor fecal microbiota to fluconazole-treated mice reconstructed the fungal community (Fig. S3E, F), but did not alter bacterial diversity (Fig. S3G, H). In summary, these results indicate that fungal reconstruction promotes diet-induced obesity in mice lacking a mycobiome.

### Fungal communities are associated with the host obese phenotype

**Fig. 3:**
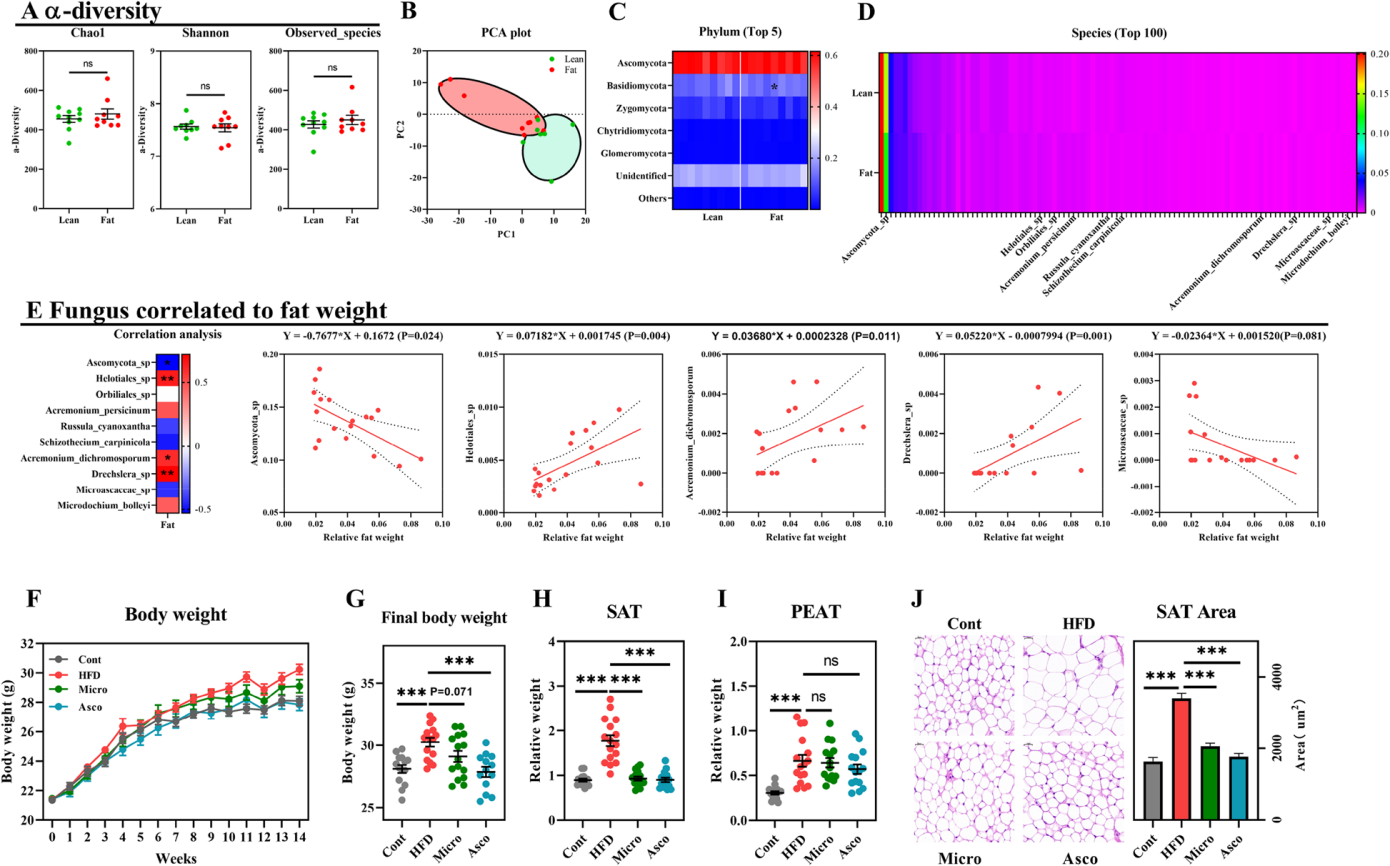
Fungal communities are associated with host obese phenotype. **A**–**D** α-Diversity (Chao1, Shannon, and observed species) (**A**), beta-diversity (PCA plot) (**B**), the top 5 fungal phyla (**C**), and the top 100 fungal genres (**D**) between obese (31.9 ± 1.29 g) and lean (30.02 ± 0.96 g) mice. C57BL/6 mice (7 weeks old, male) fed HFD for 12 weeks, and the half mice with higher body weight gain were grouped as fat; the remaining mice with lower body weight gain were named lean subjects (*n* = 9). A total of 10/100 differentiated fungal genres were marked with names. **E** Five fungus correlated to fat content through Pearson correlation and regression analysis. **F**–**J** Body weight (**F**), final body weight (**G**), the relative weight of SAT (**H**) and PEAT (**I**), and the adipocyte area of SAT (HE staining, × 400) in fungus-treated HFD mice. HFD mice (7 weeks old, male, C57BL/6) were colonized with fungus *Ascomycota_*sp. (Asco) and *Microascaceae_*sp. (Micro) for 14 weeks to test the role of fat weight-related fungi in HFD-induced obesity (*n* = 13–16). Differences among the groups were compared using Student’s *t*-test. *p < 0.05; **p < 0.01; ***p < 0.001; ns *p* > 0.05

To examine changes in obesity related to the gut fungal community, we compared fecal fungi composition between lean and obese phenotypes in HFD-fed mice. We observed that although *α*-diversity was not altered (Chao1, Shannon, and observed species levels), the PCA plot showed an obvious difference between lean and obese phenotypes (Fig. 3A and B). At the phylum level, Ascomycota was the most abundant fungus (59%), followed by Basidiomycota (12%) and was enhanced in obese mice compared to lean mice (Fig. 3C). Among top 100 fungi, 10 species were differentiated between lean and obese mice, including *Ascomycota_sp*, *Helotiales_sp*, *Orbiliales_sp*, *Acremonium_persicinum*, *Russula_cyanoxantha*, *Schizothecium_carpinicola*, *Acremonium_dichromosporum*, *Drechslera*_*sp*, *Microascaceae_sp*, and *Microdochium_bolleyi* (Fig. 3D and E; Fig. S4A). Next, we performed two-step analyses to identify fungi associated with obesity. First, we used Pearson correlation analyses between the sum of adipose tissues and 10 fungi that were significantly different between lean and obese mice. Then, for correlations identified at a *P*-value < 0.05, we conducted a regression analysis to calculate a linear equation between each fungus and fat deposition. Five associations were identified, including negative correlations with *Ascomycota_*sp. and *Microascaceae_*sp. and positive correlations with *Helotiales_sp*, *Acremonium_dichromosporum*, and *Drechslera_*sp. (Fig. 3E).

*Sporobolomyces lactosus* from *Ascomycota_*sp. (Asco) and *Microascus trigonosporus* from *Microascaceae_*sp. (Micro) were used to test the hypothesis that negatively correlated fungi improve diet-induced obesity. Colonization of Asco and Micro in mice for 14 weeks reduced body weight (Fig. 3F and G). The relative weight of SAT but not PEAT also decreased in Asco- and Micro-colonized mice (Figs. 3H and I), and Asco-colonized mice showed improvements in serum lipid content (Fig. S4B and C). Characterization of the adipose phenotypes in the SAT and liver revealed reductions in adipose size and content in fungus-treated mice but not HFD-fed mice (Fig. 3J, Fig. S4D). Furthermore, we found that administration of the oral fungus improved the expression of glucose metabolism-related genes in HFD-fed mice (Fig. S4E).

Next, we investigated whether fungal colonization ameliorated diet-induced obesity. Mice fed a HFD for 20 weeks were colonized with Asco and Micro for 14 weeks. Micro fungus-treated mice showed an improvement in body weight and SAT weight, while Asco reduced the weight of SAT and PEAT (Fig. S4F–I). Although serum CHOL levels were not altered, both Asco and Micro treatments improved serum LDL and adipocyte size and area of SAT and PEAT in obese mice (Fig. S4J–O). Therefore, gut fungal communities are associated with the host obese phenotype, and specific fungi negatively correlated with fat weight may serve as a novel biomarker for preventing and treating obesity.

Gut fungi closely associate with bacteria to regulate host metabolism [25, 26]; thus, we cannot exclude the possibility that altered gut bacterial compositions caused by antifungal drug treatment contributed to the anti-obese effect in the current study. However, fluconazole treatment significantly reduced fungal DNA abundance in feces without altering bacterial abundance (Fig. S5A). Furthermore, 16S rDNA sequencing of the fecal bacterial community revealed no changes in *α*-diversity (as assessed by the Shannon and Simpson indexes) (Fig. S5B), whereas differences in *β*-diversity were observed between fluconazole-treated and control mice (Fig. S5C). Moreover, we did not observe significance alterations in the top 10 phyla (Fig. S5D) nor in the Firmicutes/Bacteroidetes ratio (a microbial marker of obesity) in fluconazole-treated mice, indicating an independent role of gut bacteria (Fig. S5E). Although 7/30 genres were altered in response to oral antifungal drugs (Fig. S5F), bacterial function PICRUSt analysis indicated that fungal deficiency did not affect lipid metabolic KEGG pathways (data not shown). Future studies should aim to validate the role of gut bacteria in fungi-mediated lipid metabolism.

### Clec7a-deficient mice are resistant to diet-induced obesity

**Fig. 4:**
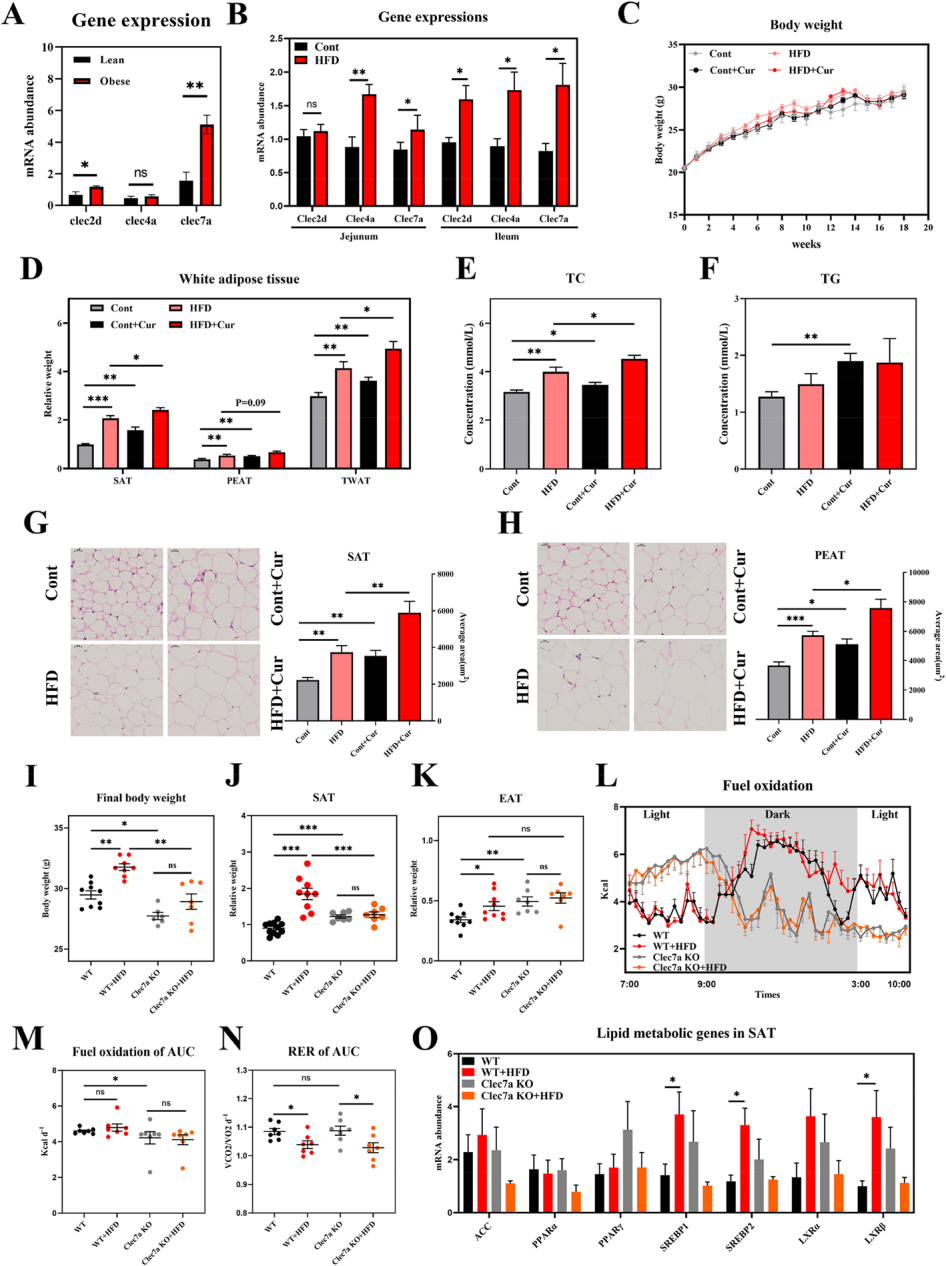
Clec7a-deficient mice are resistance to diet-induced obesity. **A** Intestinal gene expressions of *Clec2d*, *Clec4a*, and *Clec7a* in lean and obese mice fed HFD via RT-PCR (*n* = 8). **B** Intestinal gene expressions of *Clec2d*, *Clec4a*, and *Clec7a* in HFD-fed mice via RT-PCR (*n* = 7–11). **C**–**H** Body weight (**C**), the relative weight of white adipose tissues (**D**), serum TC (**E**), serum TG (**F**), and adipocyte area (HE staining, × 400) of SAT (**G**) and PEAT (**H**) in HFD mice treated with curdlan (Cur). C57BL/6 mice (7 weeks old, male) fed HFD or curdlan (10 mg/mouse) for 18 weeks to test the role of *Clec7a* activation in obesity (*n* = 8–16). **I**–**K** Final body weight (**I**), the relative weight of SAT (**J**), and epididymal adipose tissue (EAT) (**K**) in Clec7a KO mice. Seven-week-old-male C57BL/6 wild-type and Clec7a KO mice were fed HFD for 14 weeks (*n* = 7 or 11). **L**–**N** Metabolic parameters (O_2_, CO_2_, and energy consumption) of Clec7a KO mice (*n* = 6). **O** Lipid metabolic genes of SAT in Clec7a KO mice (*n* = 7 or 11). Differences among the groups were compared using Student’s *t*-test. *p < 0.05; **p < 0.01; ***p < 0.001; ns *p* > 0.05

CLRs are the main pathways for gut fungi recognition. We compared CLR changes in different mouse models. Increased intestinal mRNA abundances of *Clec2d* and *Clec7a* (Fig. 4A) and protein levels of dectin-1 (*Clec7a* encoding protein) were observed in obese mice (Fig. S6A). HFD-fed mice also showed higher abundances of *Clec4a* and *Clec7a* mRNA (Fig. 4B) and dectin-1 protein (Fig. S6B) in the small intestine.

*Clec7a* mainly recognizes glucans at fungal cell walls and may be mediated by gut fungi-derived lipid deposits. Curdlan, a common agonist of dectin1 [27, 28], was used to activate *Clec7a*, and accelerated lipid deposition was observed with a higher relative weight and area of white adipose tissues and serum lipids but not body weight gain (Fig. 4C–H). Next, we hypothesized that blockade of fungal recognition, by deleting the *Clec7a* gene, might confer a similar anti-obesity effect. *Clec7a*-KO mice gained similar amounts of weight when fed a control or HFD (Fig. 4I). Similar results were observed in the relative weight of SAT, EAT, and fuel oxidation curve, which were not increased in *Clec7a* KO mice in response to dietary HFD (Fig. 4J–N). Interestingly, *Clec7a*-KO mice presented a marked circadian alteration in fuel oxidation with or without a HFD (Fig. 4L). These results indicate that *Clec7a* is required for HFD-induced body weight gain, fat deposition, and energy expenditure and even for the regulation of circadian rhythm. We identified lipid metabolic genes in the SAT and found that these genes were not altered in HFD-fed *Clec7a* KO mice (Fig. 4O).

### Clec7a deficiency blocks the effect of gut fungus on lipid accumulation

**Fig. 5:**
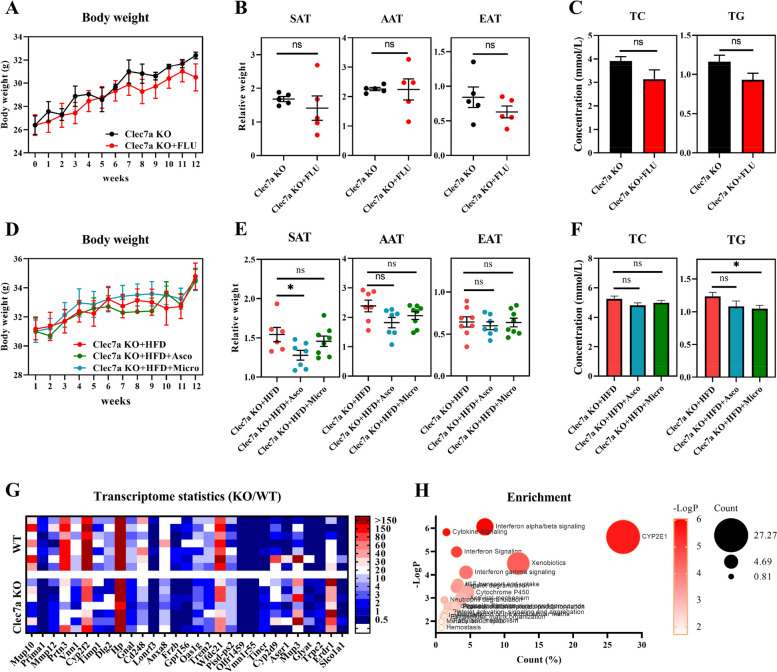
Clec7a deficiency blocks the effect of gut fungus on lipid accumulation. **A**–**C** Body weight (**A**), the relative weight of adipose tissues (**B**), and serum TC and TG (**C**) of fluconazole (FLU)-treated Clec7a KO mice. C57BL/6 *Clec7a* KO mice (15 weeks old, male) were fed HFD and treated with or without fluconazole (FLU) for 12 weeks (*n* = 5). **D**–**F** Body weight (**D**), the relative weight adipose tissues (**E**), and serum TC and TG (**F**) in fungus-treated Clec7a KO mice. HFD-fed *Clec7a* KO mice (31.1 ± 0.1 g) were orally administrated with fungus *Ascomycota_*sp. (Asco) and *Microascaceae_*sp. (Micro) for 12 weeks (*n* = 6–8). **G** Twenty-nine differentiated genes between *Clec7a* KO and wild mice by transcriptome analysis (*n* = 7). **H** KEGG pathway analysis of the 29 differentiated genes. Differences among the groups were compared using Student’s *t*-test. *p < 0.05; **p < 0.01; ***p < 0.001; ns *p* > 0.05

To examine the role of *Clec7a* in gut fungus-mediated lipid metabolism, we explored the obese phenotypic responses to antifungal drugs in HFD-fed *Clec7a* KO mice. Inconsistent with the wild-type mice (Fig. 1), gut fungi-deficient *Clec7a* KO mice (fluconazole treated for 12 weeks) did not exhibit reduced body weight gain (Fig. 5A). Similar results were also observed in the relative weight of adipose tissues (SAT, AAT, and EAT) and in serum lipids (TC and TG) (Fig. 5B and C). The role of *Clec7a* in fungus-mediated lipid metabolism was further confirmed in Asco- and Micro fungus-treated *Clec7a* KO mice, whose body weight was not markedly affected after 12 weeks of colonization (Fig. 5D). The relative weight of SAT was reduced in *Clec7a* KO mice treated with Asco fungus but not in mice colonized with Micro fungus (Fig. 5E). Both AAT and EAT were not changed in *Clec7a* KO mice (Fig. 5E and F). Colonization of Asco and Micro did not affect the AAT and EAT weights or serum TC concentrations (Fig. 5E and F), but TG was markedly reduced in Micro fungus-treated *Clec7a* KO mice (Fig. 5F). These results suggest that *Clec7a* deficiency, at least in part, blocks the anti-obese effect of gut fungus in diet-induced obesity.

To gain further insight into the potential mechanisms by which the *Clec7a* gene drives host anti-obese physiology, intestinal levels of 51,895 mRNA were profiled by transcriptome sequencing in *Clec7a* KO mice. Twenty-nine genes were identified as differentially expressed between *Clec7a* KO and wild-type mice, with 18 downregulated and 11 upregulated (|log2(fold change)|> 1.5, *p*-value < 0.5) (Fig. 5G). We then explored Reactome Gene Sets, and identified 23 pathways that were enriched, including those related to interferon-alpha/beta signaling, cytokine signaling in the immune system, CYP2E1 reactions, interferon signaling, xenobiotics, and interferon-gamma signaling (Fig. 5H). However, the specific role of these inflammatory pathways in *Clec7a*-mediated lipid metabolism needs further study.

## Discussion

Obese patients are typically classified as having the Bacteroides 2 (Bact2) enterotype, which correlates with body mass index [29]. Our previous study showed bacterial dysbiosis in HFD-fed mice characterized by alterations in biodiversity and composition [30, 31]. Obesity is characterized by altered viral taxonomic composition and weakened viral-bacterial correlations compared with lean controls [32]. Additionally, differences in the fecal mycobiome are observed between obese patients and nonobese subjects [17]. However, until now, direct evidence supporting the role of gut fungi in obesity has not been established. Our study is the first to report a marked difference in fungal communities in two obese pig models, suggesting a role of gut fungi in lipid metabolism. However, we cannot exclude the influence of genetic factors in the different animal breeds used in this study.

Germ-free animals are resistant to diet-induced obesity [23, 24]. We previously reported that bacterial dysbiosis induced in mice administered an antibiotic cocktail maintained a lean phenotype after consuming a Western-style, high-fat, sugar-rich diet [30]. Antifungal antibiotics are also reported to effectively inhibit obesity and its related disorders [33]. In the current study, disruption in the healthy intestinal fungal community was induced by fluconazole, a common antifungal agent [21]. Short-term pretreatment or long-term exposure to antifungals protected against diet-induced obesity and improved glucose hemostasis and energy expenditure, while reassembly of the fungal community by cohousing or fecal microbiota transplantation maintained diet-induced fat deposition. Altogether, these data support our hypothesis that steady-state fungal populations of the gut are directly or indirectly involved in diet-induced obesity.

A substantial body of literature demonstrates that intestinal bacterial populations can both positively and negatively influence the development and treatment of obesity [6, 34–37]. Whether the anti-obese effects we observed following antifungal drug treatment were due to primary alterations in the fungal community or were due to secondary effects on bacterial populations is unclear. Consisent with previous studies [21, 38], we also found that antifungal treatment does not alter bacterial diversity and total bacterial count, and does not change the Firmicutes/Bacteroidetes ratio, a microbial maker of obesity [39], in fluconazole-treated mice. We identified 2 fungi that were negatively correlated (*Ascomycota_*sp. and *Microascaceae_sp*) and 3 fungi that were positively correlated (*Helotiales_sp*, *Acremonium_dichromosporum*, and *Drechslera_sp*) with fat content. A study reported that gut commensal fungi (such as *Candida parapsilosis*) promote diet-induced obesity by increasing free fatty acids in the gut due to the production of fungal lipases [40]. Given this, we focused on whether *Ascomycota_*sp. and *Microascaceae_*sp. improve the metabolic phenotype in diet-induced obesity. Interestingly, Asco and Micro colonization resulted in a substantial reduction in body weight gain and fat deposition in HFD-fed mice. Given these observations, we predicted that fungal treatment would be protective in the context of obesity.

A potential link mediating the relationship between gut fungi and obesity may be the *Clec7a* receptor, which is expressed on intestinal surfaces. A study found that HFD-fed *Clec7a* KO mice had improved glucose tolerance and insulin sensitivity [20]. This is consistent with our observation that a HFD did not result in more body weight gain, fat deposition, or energy expenditure in *Clec7a*-KO mice. Interestingly, *Clec7a* deficiency also blocked the effect of gut fungi on fat deposition. The data presented here indicate that *Clec7a* is required for the occurrence of gut fungus-mediated diet-induced obesity. Fungal recognition by *Clec7a* triggers multiple downstream pathways (Raf‐1 and Syk/CARD9) to induce inflammatory or metabolic responses [41–43]. We also observed that various inflammatory pathways were enriched in *Clec7a*-KO mice. Future studies should aim to determine how *Clec7a* interacts with the inflammatory system and how the interaction between *Clec7a* and inflammatory pathways modulates lipid metabolism.

## Conclusions

In conclusion, fungal clearance ameliorated obesity induced by a high-fat diet in mice, and the obese phenotype of these mice was maintained by reconstructing the fungal community in the gut. *Clec7a* was involved in the recognition of *Ascomycota_*sp. and *Microascaaceae_*sp. in mice, and *Clec7a*-deficient mice developed resistance to diet-induced obesity and blocked the anti-obesity effects of antifungal drugs and fungi. Overall, these results suggest that intestinal fungi/*Clec7a* signaling is involved in diet-induced obesity and may have therapeutic significance as a biomarker for metabolic disorders in humans. However, the interactions between gut fungi and the downstream signaling pathways of *Clec7a* need further investigation.

## Methods

### Animal studies

All procedures were performed according to institutional guidelines and were approved by the Institutional Animal Care and Use Committee of Hunan Agricultural University. Five obese Shaziling pigs (male, 150 days old) and 5 lean control Yorkshire pigs (male, 150 days old) were selected from two different pig farms for experiments involving fecal fungal analysis [44]. Fatty Ningxiang pigs (male, 20-kg body weight) and lean DLY (male, 20-kg body weight) were raised at the same pig farm and fed the same diet for 14 weeks for fecal fungal analysis. Wild-type C57BL/6 J (Hunan Slake) and *Clec7a* KO (Cyagen company) mice were also used in this study. Fungal deficiency was induced by oral administration of 0.5 g/L fluconazole (Aladdin) in drinking water. All animals were acclimatized to the animal research facility for at least 1 week commencing with the studies. Mice were housed in a mouse facility with a 12:12 h light–dark cycle maintained at 20–22 °C. Murine diets were formulated and optimized according to our previous reports [30].

### Obesity induction

Obesity was induced by a high-fat diet (HFD) containing 52.7% corn, 5.6% casein, 18.0 soybean meal, 6.5% beer yeast, 1.4% fish meal, 0.5% salt, 1.0% premix, 11.3% lard oil, 1.5% cholesterol, and 1.5% white sugar. The breakdown of nutrient composition was as follows: 14.1% crude fat and 21.2% crude protein. The mice were fed for at least 12 weeks to induce obesity. The standard chow was formulated with 67.3% corn, 5.2% casein, 17.0% soybean meal, 6.1% beer yeast, 1.4% fish meal, 0.5% salt, 1.0% premix, 0.7% lard oil, and 0.8% soybean oil (containing 5.0% crude fat and 21.4% crude protein).

### Mouse cohousing

Six-week-old mice were pretreated with fluconazole for 1 week. Then, fluconazole-pretreated mice were cohoused with control or obese mice (HFD feeding for 20 weeks) in a cage. All mice were fed a HFD and consumed food and water ad libitum.

### Fecal microbial transplantation (FMT)

Seven-week-old male C57BL/6 mice received 1 week of fluconazole and were then fed a HFD (PreFlu + HFD). Five healthy mice were selected as microbial donors, and fecal samples were collected and resuspended in normal saline (feces/normal saline = 1:5, w/v). Fecal samples were mixed and centrifuged at 1000 × g for 3 min, and the supernatants were transplanted into the PreFlu + HFD-fed mice by gavaging with 0.2 mL per mouse once daily for the first week and then challenged twice weekly for 12 or 15 weeks.

### Metabolic parameter analysis

The peripheral response to glucose availability was assessed by a glucose tolerance test (GTT) in mice fasted for 12 h and injected with 1 g/kg glucose. The insulin response was examined by an insulin tolerance test (ITT) after fasting mice for 6 h and injecting 0.75 U/kg insulin. Orbital bleeding was conducted, and serum samples were separated by centrifugation at 1500 g for 10 min at 4 °C to obtain measures for triglycerides (TG), cholesterol (CHOL), high‐density lipoprotein (HDL), low‐density lipoprotein (LDL), and glucose using a Cobas c‐311 Coulter Chemistry analyzer.

Metabolic parameters (O_2_, CO_2_, and energy consumption) were recorded in an International FOXBOX™ field oxygen analysis system for 48 h, and data from a continuous 48-h period were calibrated using the body weight for the fuel oxidation and respiratory exchange ratio (RER) [45].

### Gene expression

Gene expression quantification was performed by real-time PCR. Total RNA was isolated from intestinal and adipose tissues that were frozen and ground in liquid nitrogen with TRIzol reagent (Invitrogen, Carlsbad, CA, USA) and then treated with DNase I (Invitrogen). Reverse transcription was conducted at 37 °C for 15 min and 95 °C for 5 s. The primers used in this study were designed according to the mouse sequence (Table S1). A comparative relationship between reaction cycles (CT) was used to determine gene expression relative to β‐actin control (housekeeping gene).

Fecal DNA was extracted using a DNA extraction kit (Seno Biotechnologies Co., Ltd.), and RT-PCR was performed using 16S primers (F: AGAGTTTGATCMTGGCTCAG, R: CTGCTGCCTYCCGTA) and ITS primers (F: ATTGGAGGGCAAGTCTGGTG, R: CCGATCCCTAGTCGGCATAG), followed by quantification of the PCR products by fluorescence using SYBR Green.

### Hematoxylin eosin (HE) and Oil Red O staining

Adipose tissues and liver were fixed in 4% paraformaldehyde, paraffin embedded, and sectioned at 5–7 μm. HE and Oil Red O staining were conducted according to standard methods. For adipocytes size, cells were outlined by enhanced contrast function, and the calculated area was obtained, in pixels, for each with ImageJ.

### Microbial sequencing

Total DNA was isolated from the fecal samples using a DNeasy PowerSoil kit (Qiagen, Hilden, Germany). For fungal diversity analysis, the ITS1 variable regions were amplified using universal primer pairs (ITS1F: 5′-CTTGGTCATTTAGAGGAAGTA-3′; ITS2: 5′-GCTGCGTTCTTCATCGATGC-3′). For bacterial diversity analysis, 16S rDNA genes of distinct regions (16S V3–V4) were amplified using specific primers (515F-806R) with barcodes. Sequencing was performed on an Illumina MiSeq with two paired-end read cycles of 300 bases each. Clean reads were subjected to primer sequence removal and clustering to generate operational taxonomic units (OTUs) using VSEARCH software with a 97% similarity cutoff. The representative read of each OTU was selected using the QIIME package. All representative reads were annotated and blasted against the UNITE database using BLAST.

### Fungal strains and treatment

The *Sporobolomyces lactosus* and *Microascus trigonosporus* strains used in this study were purchased from Beijing Beina Biotechnology Institute (Beijing, China). Unless otherwise stated, *Sporobolomyces lactosus* were grown in YM Broth (YMB, Qingdao Hope Biotechnology) and YM agar (YMA) plates at 30 °C, pH = 6.2 ± 0.2. *Microascus trigonosporus* was grown in malt extract broth (MEB, malt extract, glucose, peptone and distilled water) and ME agar (MEA) plates at 30 °C. Mice received mono-colonization (10^9^ cfu) for 7 continuous days and then twice per week in later weeks.

### Curdlan administration

Curdlan (Shanghai Macklin) was used to test the role of dectin1 activation in HFD-induced obese mice [20]. Seven-week-old C57BL/6 mice (male) were orally gavaged with curdlan (50 mg/mL, 0.2 mL/mouse) daily for the first week and then twice weekly for the next 17 weeks. All mice consumed food and water ad libitum.

### Statistical analysis

All values are given as means ± SEM. Differences among the groups were compared using Student’s *t*-test. All statistical analysis were performed using GraphPad Prism 9 software, and the differences were considered significant when *p* < 0.05.

### Supplementary Information

**Additional file 1: Supplemental Fig. 1.** Differences in gut fungi in obese animal models. (A) Fungal diversity between obese Shaziling (SZL) pigs and lean Yorkshire pigs (*n*=5); (B) Fungal diversity between obese Ningxiang (NX) pigs and lean DLY pigs (*n*=7 or 8); (C) Fungal diversity in HFD fed mice (*n*=7); (D) Fungal phyla and makers (genus) in obese Shaziling (SZL) pigs (*n*=5); (E) Fungal phyla and makers (genus) in Ningxiang (NX) pigs (*n*=7 or 8); (F) Fungal phyla and makers (genus) in HFD fed mice (*n*=5). Differences among the groups were compared using Student’s t test. *p<0.05; ***p<0.001. **Supplemental Fig. 2.** Fungi deficiency protects mice against diet-induced obesity. (A-F) Body weight (A), final body weight (B), the relative weight of SAT (C), AAT (D), PEAT (E), and white adipose tissue enlargement in fluconazole (Flu) treated male mice. 7 weeks old-male C57BL/6 mice were treated with fluconazole (Flu) for 16 weeks (*n*=8); (G, H) Western blot of dectin1 expression (*n*=3); (I-M) Body weight (I), the relative weight of SAT (J), total white adipose tissue (TWAT) (K), serum TC (L), and HDL (M) in fluconazole (Flu) treated female mice (*n*=10). 6-7 weeks old-female C57BL/6 mice were treated with fluconazole (Flu) lasted for 16 weeks to test the role of gut fungi in different sexes (*n*=8-10). Differences among the groups were compared using Student’s t test. *p<0.05; **p<0.01; ***p<0.001; ns p>0.05. **Supplemental Fig. 3.** Fecal microbial compositions in FMT and cohoused mice. (A-D) α-diversity (A) and β-diversity (B) of gut fungi in cohoused with control mice, α-diversity (C) and β-diversity (D) of gut fungi in cohoused with obese mice (*n*=6); (E-H) α-diversity (E) and β-diversity (F) of gut fungi in cohoused with with obese mice, α-diversity (G) and β-diversity (H) of gut bacteria in FMT mice (*n*=8). Differences among the groups were compared using Student’s t test. *p<0.05; **p<0.01; ***p<0.001; ns *p*>0.05. **Supplemental Fig. 4.** Fungal communities are associated with the host obese phenotype. (A) 10 differentiated genres from Fig.3C between obese and lean mice; (B-D) Serum TC (B), LDL (C), and liver lipid in fungus treated HFD mice (*n*=14 or 16). HFD mice (7 week old, male, C57BL/6) were colonized with fungus Ascomycota_sp (Asco) and Microascaceae_sp (Micro) for 14 weeks to test the role of fat weight-related fungi in HFD-induced obesity (n=13-16). The liver lipid was stained with oil O and the redder indicated more lipid depositions; (E) Glucose metabolism gene expression with fungus supplementation (*n*=8); (F-O) Body weight (F), final body weight (G), the relative weight of SAT (H) and PEAT(II), serum TC (J) and LDL (K), and adipocyte area of SAT and PEAT (L-O) of fungus treated obese mice. Obese mice (male, C57BL/6, 39.1±0.1g ) were induced by feeding HFD for 20 weeks (fat), then replaced by the standard chow and challenged with two fungi for 14 weeks (*n*=9-10). Differences among the groups were compared using Student’s t test. *p<0.05; **p<0.01; ***p<0.001; ns *p*>0.05. **Supplemental Fig. 5.** Fecal bacterial composition in fungi-deficient mice. (A) Fecal DNA of bacteria and fungi with RT-PCR in fungi-deficient mice (*n*=8); (B-F) a-diversity (B), β-diversity (C), top 10 phyla (D), Firmicutes/Bacteroidetes ratio (E),top 30 genre (F) in fungi-deficient mice. Fecal bacterial compositions of HFD (feeding 12 weeks) and fluconazole (Flu) treated mice were sequenced by 16S rDNA (n=12-13). Differences among the groups were compared using Student’s t test. *p<0.05; **p<0.01; ***p<0.001; ns *p*>0.05. **Supplemental Fig. 6.** Dectin-1 expression in obese mice. (A) Western blot in Lean and Obese mice fed HFD (*n*=3); (B) Western blot in HFD fed mice (*n*=3). Differences among the groups were compared using Student’s t test. *p<0.05; **p<0.01; ***p<0.001; ns *p*>0.05.

**Additional file 2: Supplemental Table 1.** Study primers.

## Data Availability

The data supporting the conclusions of this article are available in the NCBI Sequence Read Archive (SRA) repository: PRJNA792578, PRJNA796156, PRJNA781286, PRJNA810881, PRJNA810904, PRJNA1013033, PRJNA 1013613, PRJNA 1020983, and PRJNA 1022997.
