# Supplementary figures and images for: Clec7a drives gut fungus-mediated host lipid deposition
Source: Microbiome. 2023 Nov 25;11:264. doi: 10.1186/s40168-023-01698-5 (PMC10675981; doi:10.1186/s40168-023-01698-5)

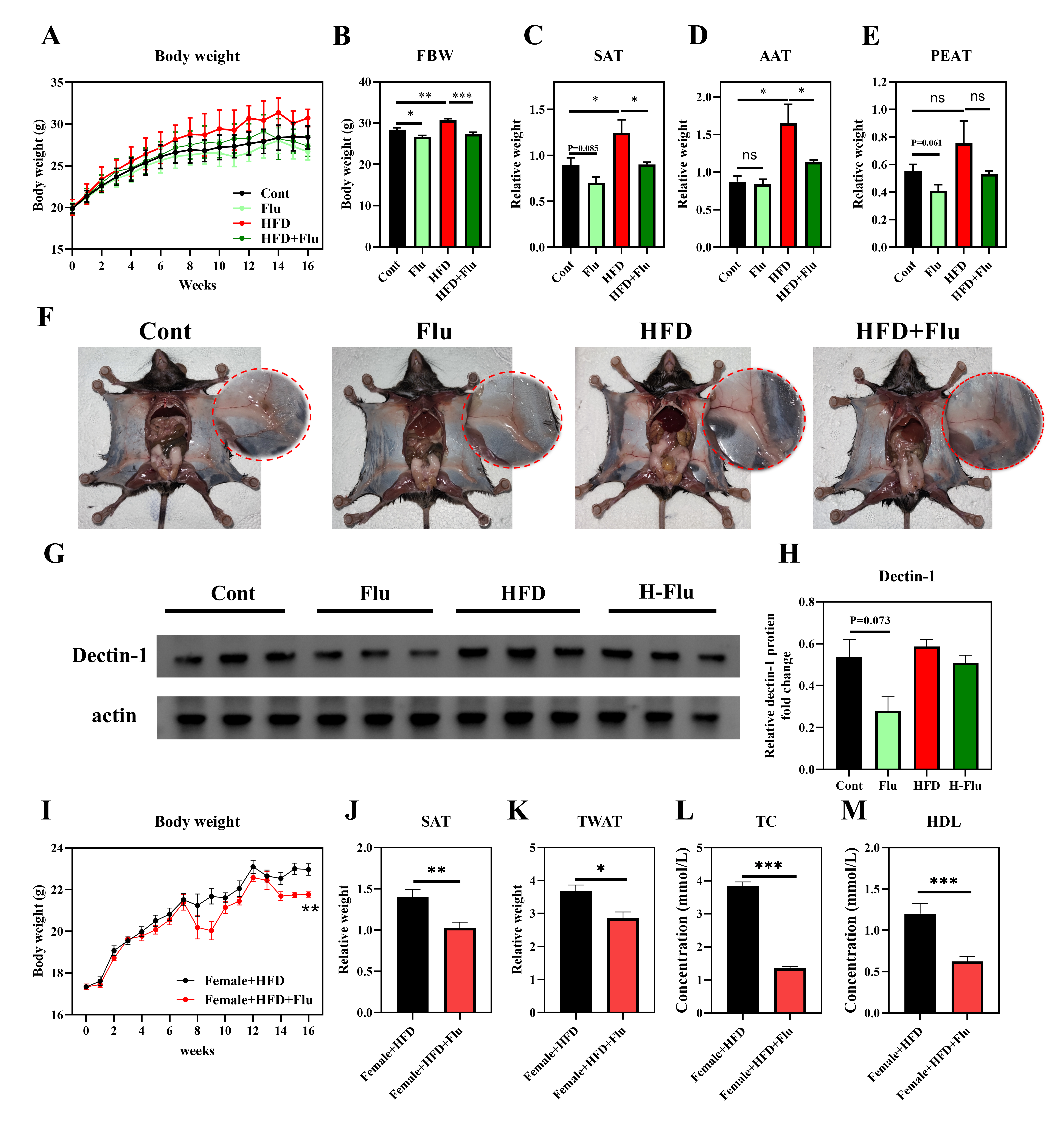

Supplement: Supplementary file 2 — Additional file 1: Supplemental Fig. 1. Differences in gut fungi in obese animal models. (A) Fungal diversity between obese Shaziling (SZL) pigs and lean Yorkshire pigs (n=5); (B) Fungal diversity between obese Ningxiang (NX) pigs and lean DLY pigs (n=7 or 8); (C) Fungal diversity in HFD fed mice (n=7); (D) Fungal phyla and makers (genus) in obese Shaziling (SZL) pigs (n=5); (E) Fungal phyla and makers (genus) in Ningxiang (NX) pigs (n=7 or 8); (F) Fungal phyla and makers (genus) in HFD fed mice (n=5). Differences among the groups were compared using Student’s t test. *p<0.05; ***p<0.001. Supplemental Fig. 2. Fungi deficiency protects mice against diet-induced obesity. (A-F) Body weight (A), final body weight (B), the relative weight of SAT (C), AAT (D), PEAT (E), and white adipose tissue enlargement in fluconazole (Flu) treated male mice. 7 weeks old-male C57BL/6 mice were treated with fluconazole (Flu) for 16 weeks (n=8); (G, H) Western blot of dectin1 expression (n=3); (I-M) Body weight (I), the relative weight of SAT (J), total white adipose tissue (TWAT) (K), serum TC (L), and HDL (M) in fluconazole (Flu) treated female mice (n=10). 6-7 weeks old-female C57BL/6 mice were treated with fluconazole (Flu) lasted for 16 weeks to test the role of gut fungi in different sexes (n=8-10). Differences among the groups were compared using Student’s t test. *p<0.05; **p<0.01; ***p<0.001; ns p>0.05. Supplemental Fig. 3. Fecal microbial compositions in FMT and cohoused mice. (A-D) α-diversity (A) and β-diversity (B) of gut fungi in cohoused with control mice, α-diversity (C) and β-diversity (D) of gut fungi in cohoused with obese mice (n=6); (E-H) α-diversity (E) and β-diversity (F) of gut fungi in cohoused with with obese mice, α-diversity (G) and β-diversity (H) of gut bacteria in FMT mice (n=8). Differences among the groups were compared using Student’s t test. *p<0.05; **p<0.01; ***p<0.001; ns p>0.05. Supplemental Fig. 4. Fungal communities are associated with the host ob [file 40168_2023_1698_MOESM1_ESM.zip › Figure S2.tif]

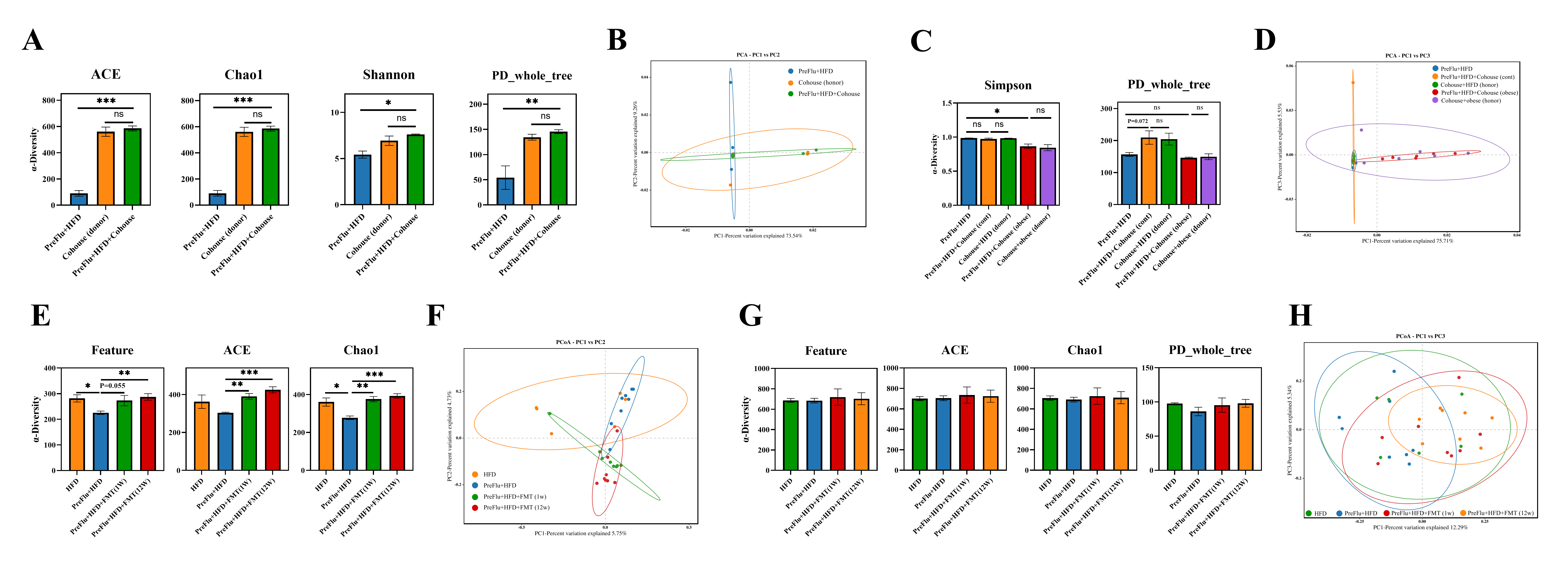

Supplement: Supplementary file 2 — Additional file 1: Supplemental Fig. 1. Differences in gut fungi in obese animal models. (A) Fungal diversity between obese Shaziling (SZL) pigs and lean Yorkshire pigs (n=5); (B) Fungal diversity between obese Ningxiang (NX) pigs and lean DLY pigs (n=7 or 8); (C) Fungal diversity in HFD fed mice (n=7); (D) Fungal phyla and makers (genus) in obese Shaziling (SZL) pigs (n=5); (E) Fungal phyla and makers (genus) in Ningxiang (NX) pigs (n=7 or 8); (F) Fungal phyla and makers (genus) in HFD fed mice (n=5). Differences among the groups were compared using Student’s t test. *p<0.05; ***p<0.001. Supplemental Fig. 2. Fungi deficiency protects mice against diet-induced obesity. (A-F) Body weight (A), final body weight (B), the relative weight of SAT (C), AAT (D), PEAT (E), and white adipose tissue enlargement in fluconazole (Flu) treated male mice. 7 weeks old-male C57BL/6 mice were treated with fluconazole (Flu) for 16 weeks (n=8); (G, H) Western blot of dectin1 expression (n=3); (I-M) Body weight (I), the relative weight of SAT (J), total white adipose tissue (TWAT) (K), serum TC (L), and HDL (M) in fluconazole (Flu) treated female mice (n=10). 6-7 weeks old-female C57BL/6 mice were treated with fluconazole (Flu) lasted for 16 weeks to test the role of gut fungi in different sexes (n=8-10). Differences among the groups were compared using Student’s t test. *p<0.05; **p<0.01; ***p<0.001; ns p>0.05. Supplemental Fig. 3. Fecal microbial compositions in FMT and cohoused mice. (A-D) α-diversity (A) and β-diversity (B) of gut fungi in cohoused with control mice, α-diversity (C) and β-diversity (D) of gut fungi in cohoused with obese mice (n=6); (E-H) α-diversity (E) and β-diversity (F) of gut fungi in cohoused with with obese mice, α-diversity (G) and β-diversity (H) of gut bacteria in FMT mice (n=8). Differences among the groups were compared using Student’s t test. *p<0.05; **p<0.01; ***p<0.001; ns p>0.05. Supplemental Fig. 4. Fungal communities are associated with the host ob [file 40168_2023_1698_MOESM1_ESM.zip › Figure S3.tif]

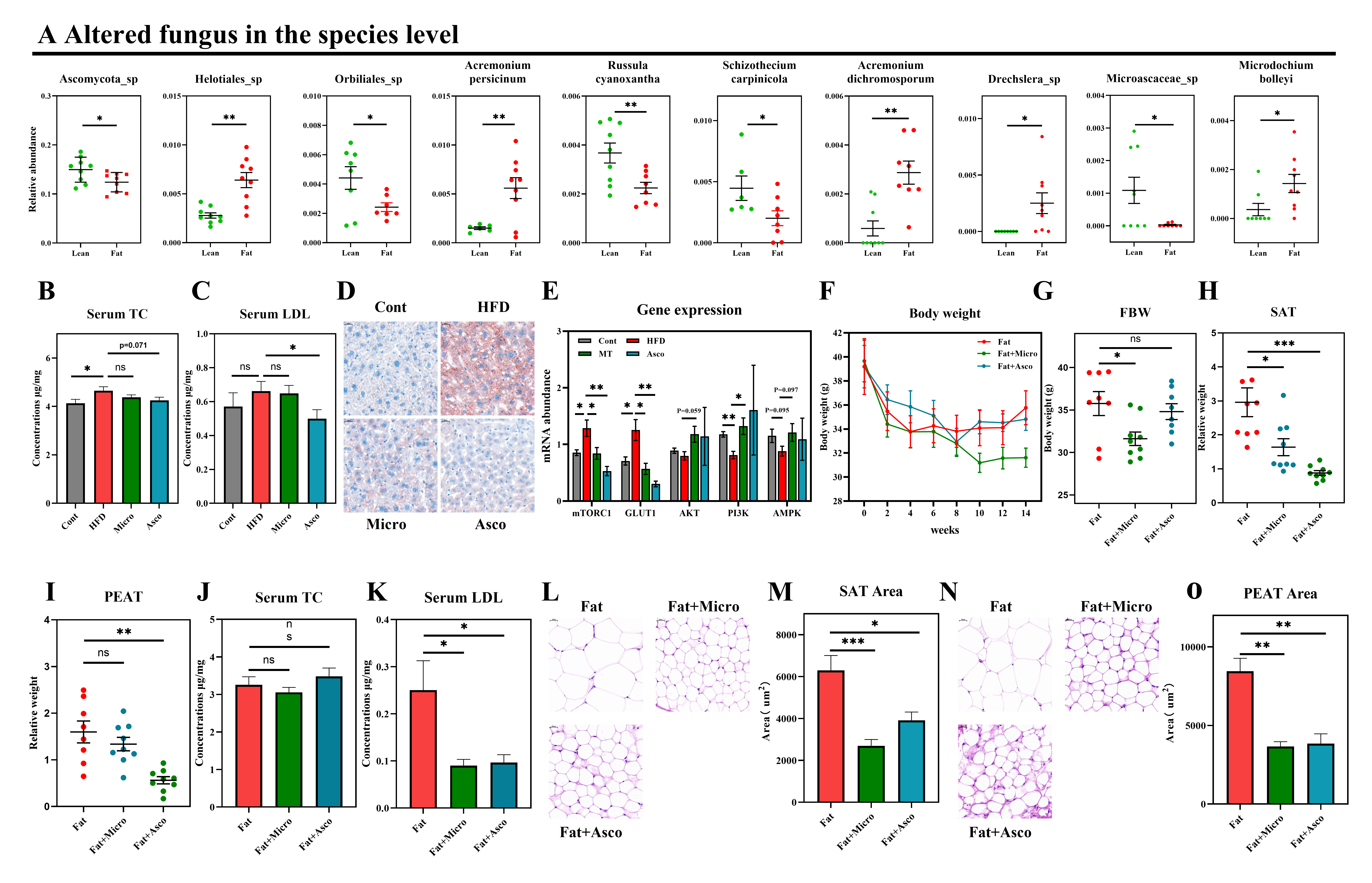

Supplement: Supplementary file 2 — Additional file 1: Supplemental Fig. 1. Differences in gut fungi in obese animal models. (A) Fungal diversity between obese Shaziling (SZL) pigs and lean Yorkshire pigs (n=5); (B) Fungal diversity between obese Ningxiang (NX) pigs and lean DLY pigs (n=7 or 8); (C) Fungal diversity in HFD fed mice (n=7); (D) Fungal phyla and makers (genus) in obese Shaziling (SZL) pigs (n=5); (E) Fungal phyla and makers (genus) in Ningxiang (NX) pigs (n=7 or 8); (F) Fungal phyla and makers (genus) in HFD fed mice (n=5). Differences among the groups were compared using Student’s t test. *p<0.05; ***p<0.001. Supplemental Fig. 2. Fungi deficiency protects mice against diet-induced obesity. (A-F) Body weight (A), final body weight (B), the relative weight of SAT (C), AAT (D), PEAT (E), and white adipose tissue enlargement in fluconazole (Flu) treated male mice. 7 weeks old-male C57BL/6 mice were treated with fluconazole (Flu) for 16 weeks (n=8); (G, H) Western blot of dectin1 expression (n=3); (I-M) Body weight (I), the relative weight of SAT (J), total white adipose tissue (TWAT) (K), serum TC (L), and HDL (M) in fluconazole (Flu) treated female mice (n=10). 6-7 weeks old-female C57BL/6 mice were treated with fluconazole (Flu) lasted for 16 weeks to test the role of gut fungi in different sexes (n=8-10). Differences among the groups were compared using Student’s t test. *p<0.05; **p<0.01; ***p<0.001; ns p>0.05. Supplemental Fig. 3. Fecal microbial compositions in FMT and cohoused mice. (A-D) α-diversity (A) and β-diversity (B) of gut fungi in cohoused with control mice, α-diversity (C) and β-diversity (D) of gut fungi in cohoused with obese mice (n=6); (E-H) α-diversity (E) and β-diversity (F) of gut fungi in cohoused with with obese mice, α-diversity (G) and β-diversity (H) of gut bacteria in FMT mice (n=8). Differences among the groups were compared using Student’s t test. *p<0.05; **p<0.01; ***p<0.001; ns p>0.05. Supplemental Fig. 4. Fungal communities are associated with the host ob [file 40168_2023_1698_MOESM1_ESM.zip › Figure S4.tif]

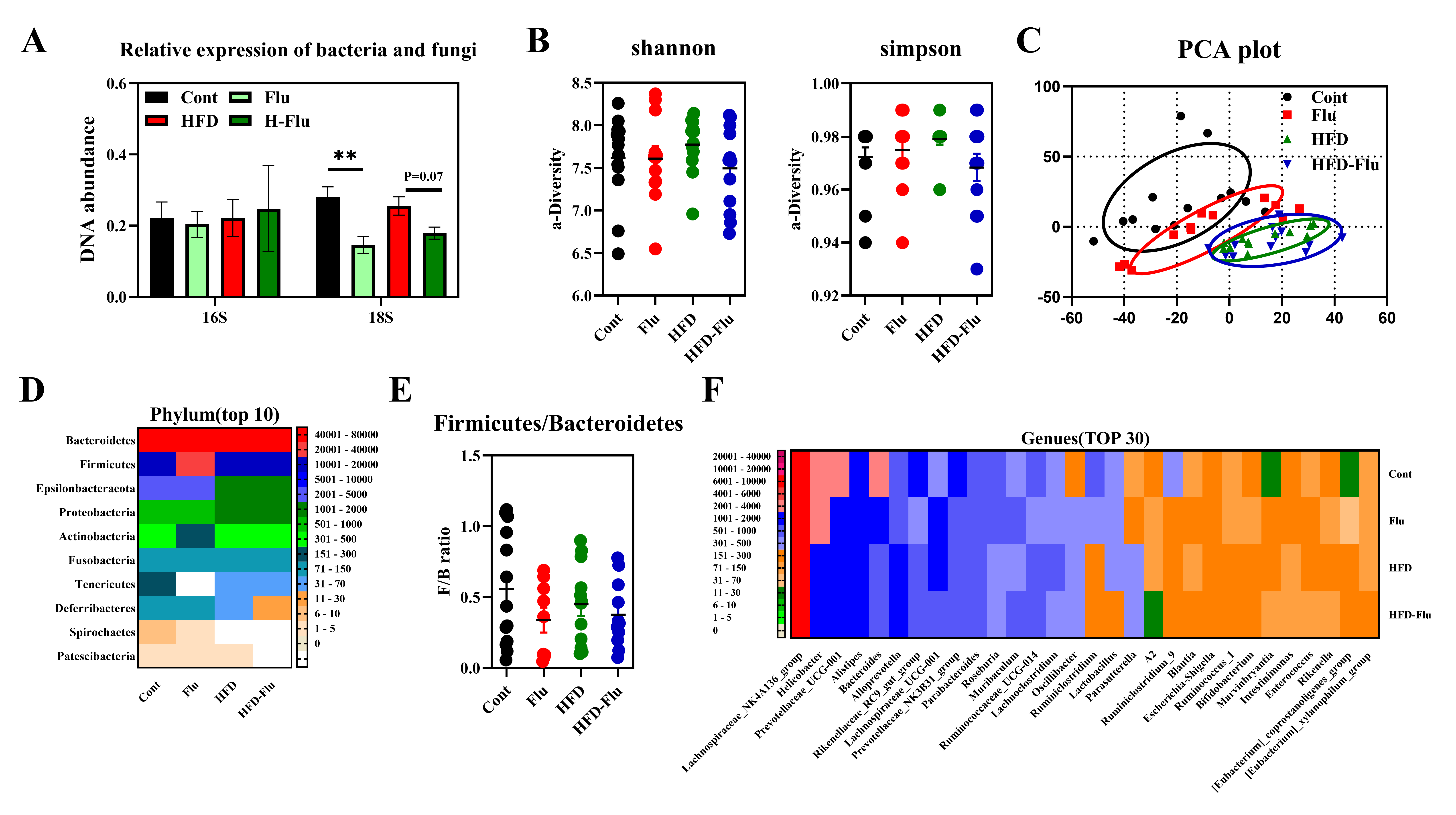

Supplement: Supplementary file 2 — Additional file 1: Supplemental Fig. 1. Differences in gut fungi in obese animal models. (A) Fungal diversity between obese Shaziling (SZL) pigs and lean Yorkshire pigs (n=5); (B) Fungal diversity between obese Ningxiang (NX) pigs and lean DLY pigs (n=7 or 8); (C) Fungal diversity in HFD fed mice (n=7); (D) Fungal phyla and makers (genus) in obese Shaziling (SZL) pigs (n=5); (E) Fungal phyla and makers (genus) in Ningxiang (NX) pigs (n=7 or 8); (F) Fungal phyla and makers (genus) in HFD fed mice (n=5). Differences among the groups were compared using Student’s t test. *p<0.05; ***p<0.001. Supplemental Fig. 2. Fungi deficiency protects mice against diet-induced obesity. (A-F) Body weight (A), final body weight (B), the relative weight of SAT (C), AAT (D), PEAT (E), and white adipose tissue enlargement in fluconazole (Flu) treated male mice. 7 weeks old-male C57BL/6 mice were treated with fluconazole (Flu) for 16 weeks (n=8); (G, H) Western blot of dectin1 expression (n=3); (I-M) Body weight (I), the relative weight of SAT (J), total white adipose tissue (TWAT) (K), serum TC (L), and HDL (M) in fluconazole (Flu) treated female mice (n=10). 6-7 weeks old-female C57BL/6 mice were treated with fluconazole (Flu) lasted for 16 weeks to test the role of gut fungi in different sexes (n=8-10). Differences among the groups were compared using Student’s t test. *p<0.05; **p<0.01; ***p<0.001; ns p>0.05. Supplemental Fig. 3. Fecal microbial compositions in FMT and cohoused mice. (A-D) α-diversity (A) and β-diversity (B) of gut fungi in cohoused with control mice, α-diversity (C) and β-diversity (D) of gut fungi in cohoused with obese mice (n=6); (E-H) α-diversity (E) and β-diversity (F) of gut fungi in cohoused with with obese mice, α-diversity (G) and β-diversity (H) of gut bacteria in FMT mice (n=8). Differences among the groups were compared using Student’s t test. *p<0.05; **p<0.01; ***p<0.001; ns p>0.05. Supplemental Fig. 4. Fungal communities are associated with the host ob [file 40168_2023_1698_MOESM1_ESM.zip › Figure S5.tif]

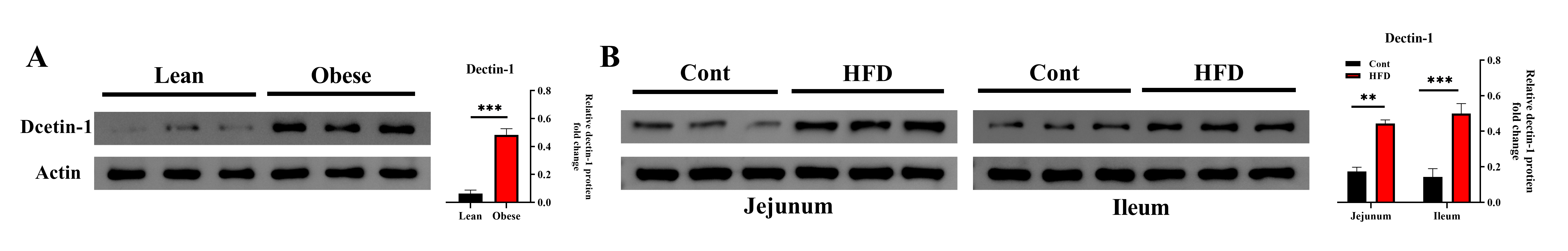

Supplement: Supplementary file 2 — Additional file 1: Supplemental Fig. 1. Differences in gut fungi in obese animal models. (A) Fungal diversity between obese Shaziling (SZL) pigs and lean Yorkshire pigs (n=5); (B) Fungal diversity between obese Ningxiang (NX) pigs and lean DLY pigs (n=7 or 8); (C) Fungal diversity in HFD fed mice (n=7); (D) Fungal phyla and makers (genus) in obese Shaziling (SZL) pigs (n=5); (E) Fungal phyla and makers (genus) in Ningxiang (NX) pigs (n=7 or 8); (F) Fungal phyla and makers (genus) in HFD fed mice (n=5). Differences among the groups were compared using Student’s t test. *p<0.05; ***p<0.001. Supplemental Fig. 2. Fungi deficiency protects mice against diet-induced obesity. (A-F) Body weight (A), final body weight (B), the relative weight of SAT (C), AAT (D), PEAT (E), and white adipose tissue enlargement in fluconazole (Flu) treated male mice. 7 weeks old-male C57BL/6 mice were treated with fluconazole (Flu) for 16 weeks (n=8); (G, H) Western blot of dectin1 expression (n=3); (I-M) Body weight (I), the relative weight of SAT (J), total white adipose tissue (TWAT) (K), serum TC (L), and HDL (M) in fluconazole (Flu) treated female mice (n=10). 6-7 weeks old-female C57BL/6 mice were treated with fluconazole (Flu) lasted for 16 weeks to test the role of gut fungi in different sexes (n=8-10). Differences among the groups were compared using Student’s t test. *p<0.05; **p<0.01; ***p<0.001; ns p>0.05. Supplemental Fig. 3. Fecal microbial compositions in FMT and cohoused mice. (A-D) α-diversity (A) and β-diversity (B) of gut fungi in cohoused with control mice, α-diversity (C) and β-diversity (D) of gut fungi in cohoused with obese mice (n=6); (E-H) α-diversity (E) and β-diversity (F) of gut fungi in cohoused with with obese mice, α-diversity (G) and β-diversity (H) of gut bacteria in FMT mice (n=8). Differences among the groups were compared using Student’s t test. *p<0.05; **p<0.01; ***p<0.001; ns p>0.05. Supplemental Fig. 4. Fungal communities are associated with the host ob [file 40168_2023_1698_MOESM1_ESM.zip › Figure S6.tif]

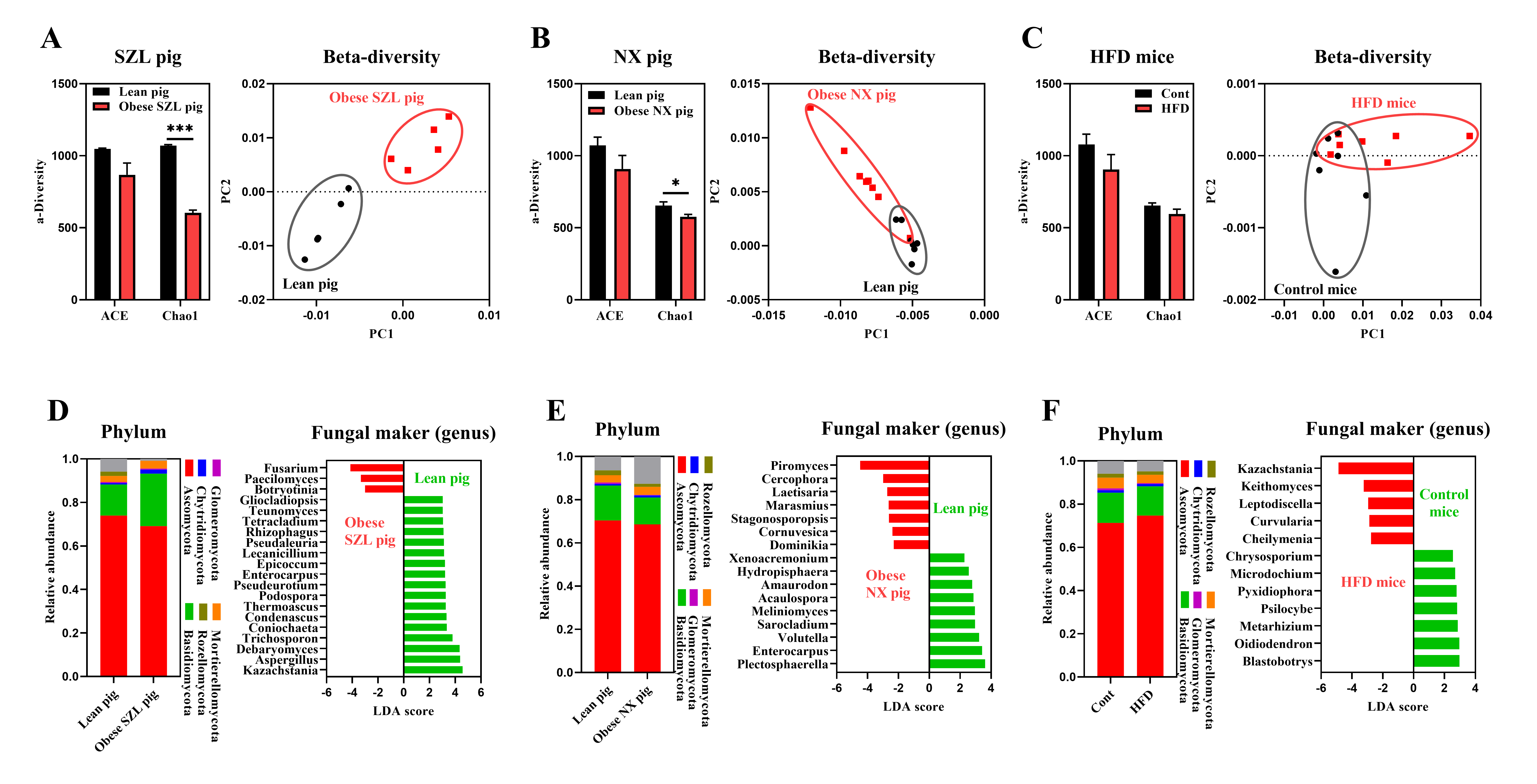

Supplement: Supplementary file 2 — Additional file 1: Supplemental Fig. 1. Differences in gut fungi in obese animal models. (A) Fungal diversity between obese Shaziling (SZL) pigs and lean Yorkshire pigs (n=5); (B) Fungal diversity between obese Ningxiang (NX) pigs and lean DLY pigs (n=7 or 8); (C) Fungal diversity in HFD fed mice (n=7); (D) Fungal phyla and makers (genus) in obese Shaziling (SZL) pigs (n=5); (E) Fungal phyla and makers (genus) in Ningxiang (NX) pigs (n=7 or 8); (F) Fungal phyla and makers (genus) in HFD fed mice (n=5). Differences among the groups were compared using Student’s t test. *p<0.05; ***p<0.001. Supplemental Fig. 2. Fungi deficiency protects mice against diet-induced obesity. (A-F) Body weight (A), final body weight (B), the relative weight of SAT (C), AAT (D), PEAT (E), and white adipose tissue enlargement in fluconazole (Flu) treated male mice. 7 weeks old-male C57BL/6 mice were treated with fluconazole (Flu) for 16 weeks (n=8); (G, H) Western blot of dectin1 expression (n=3); (I-M) Body weight (I), the relative weight of SAT (J), total white adipose tissue (TWAT) (K), serum TC (L), and HDL (M) in fluconazole (Flu) treated female mice (n=10). 6-7 weeks old-female C57BL/6 mice were treated with fluconazole (Flu) lasted for 16 weeks to test the role of gut fungi in different sexes (n=8-10). Differences among the groups were compared using Student’s t test. *p<0.05; **p<0.01; ***p<0.001; ns p>0.05. Supplemental Fig. 3. Fecal microbial compositions in FMT and cohoused mice. (A-D) α-diversity (A) and β-diversity (B) of gut fungi in cohoused with control mice, α-diversity (C) and β-diversity (D) of gut fungi in cohoused with obese mice (n=6); (E-H) α-diversity (E) and β-diversity (F) of gut fungi in cohoused with with obese mice, α-diversity (G) and β-diversity (H) of gut bacteria in FMT mice (n=8). Differences among the groups were compared using Student’s t test. *p<0.05; **p<0.01; ***p<0.001; ns p>0.05. Supplemental Fig. 4. Fungal communities are associated with the host ob [file 40168_2023_1698_MOESM1_ESM.zip › Figure S1.tif]
