# Supplementary material for: Clec7a drives gut fungus-mediated host lipid deposition
Source: Microbiome. 2023 Nov 25;11:264. doi: 10.1186/s40168-023-01698-5 (PMC10675981; doi:10.1186/s40168-023-01698-5)
Supplement: Supplementary file 3 — Additional file 2: Supplemental Table 1. Study primers. [file 40168_2023_1698_MOESM2_ESM.docx]

**Supplementary Table 1** Primers used in this study

| Gene | Forward sequence (5′-3′) | Reverse sequence (3′-5′) |
| --- | --- | --- |
| *β-Actin* | GTCCACCTTCCAGCAGATGT | GAAAGGGTGTAAAACGCAGC |
| *ACC* | ACCTGTGTGGTGGAATTTCAGT | ACATTCTGTTTAGCGTGGGGA |
| *PPARα* | GTGCAGCCTCAGCCAAGTT | TGGGGAGAGAGGACAGATGG |
| *PPARγ* | GGCTGCAGCGCTAAATTCTT | TGTGTCAACCATGGTAATTTCAGT |
| *SREBP1* | ACTTTTCCTTAACGTGGGCCT | TGAGCTGGAGCATGTCTTCG |
| *SREBP2* | GCTGTCGGGTGTCATGGG | ACAAACTGTAGCATCTCGTCGAT |
| *LXRα* | CGACAGTTTTGGTAGAGGGAC | ACTCCGTTGCAGAATCAGG |
| *LXRβ* | GCTACAACCACGAGACAGAAT | GGCGATAAGCAAGGCATACT |
| *Clec7a* | TTAGACTTCAGCACTCAAGACATC | CAGCAACCACTACTACCACAA |
| *Clec2d* | GCAGGAGGTAGAAGTGGGTAAAA | AGGTCTTGTTGACTGGGATCTG |
| *Clec4a* | CGAAGGCATCTTGGAATGAGAG | CCAGCACTTGTGTTCAGGTT |
| *mTORC1* | ACCGGCACACATTTGAAGAAG | CTCGTTGAGGATCAGCAAGG |
| *GLUT1* | CCATGTATGTGGGAGAGGTGT | TTGCCCATGATGGAGTCTAAG |
| *AKT* | AAGAAGGAGGTCATCGTCGC | CTTGAGGGCCGTAAGGAAGG |
| *PI3K* | TATTGCGAGGGAAGCGAGAC | ACTTCGCCGTCTACCACTAC |
| *AMPK* | TTCGGGAAAGTGAAGGTGGG | TCTTCTGCCGGTTGAGTATCT |
| *16S* | AGAGTTTGATCMTGGCTCAG | CTGCTGCCTYCCGTA |
| *18S* | ATTGGAGGGCAAGTCTGGTG | CCGATCCCTAGTCGGCATAG |
